# Synergistic Activity of Vancomycin and Gentamicin Against *Staphylococcus aureus* Biofilms on Polyurethane Surface

**DOI:** 10.3390/microorganisms13051119

**Published:** 2025-05-13

**Authors:** Nicolas Henrique Borges, Paula Hansen Suss, Gabriel Burato Ortis, Leticia Ramos Dantas, Felipe Francisco Tuon

**Affiliations:** Laboratory of Emerging Infectious Diseases, School of Medicine, Pontifícia Universidade Católica do Paraná, Curitiba 80215-901, Brazil; nicolashenriqueborges@hotmail.com (N.H.B.); paula.h@pucpr.br (P.H.S.); gabrielburatortis@hotmail.com (G.B.O.); leticia.dantas@pucpr.edu.br (L.R.D.)

**Keywords:** vancomycin, gentamicin, biofilm, *Staphylococcus aureus*, catheter, lock therapy

## Abstract

*Staphylococcus aureus* are frequently associated with biofilm formation on intravascular devices. Biofilms limit antimicrobial penetration and promote phenotypic resistance, challenging conventional treatment strategies. Vancomycin (VAN) and gentamicin (GEN) have been used clinically, but their combined antibiofilm activity remains underexplored. This study evaluates the efficacy of VAN and GEN, alone and in combination, against biofilms formed by methicillin-resistant *Staphylococcus aureus* (MRSA) and methicillin-sensitive *S. aureus* (MSSA) on polyurethane. MICs were determined for VAN and GEN. Biofilm biomass and metabolic activity were quantified using crystal violet and MTT assays, respectively. Biofilm viability was assessed through fluorescence microscopy and a modified Calgary Biofilm Device. A continuous-flow peristaltic model was developed to test treatment under simulated catheter conditions. While monotherapy with VAN or GEN had modest effects, their combination significantly reduced biomass and metabolic activity. VAN 20 mg/L + GEN 8 mg/L and VAN 40 mg/L + GEN 8 mg/L achieved over 70% reduction in MRSA biofilm viability and complete eradication in MBEC assays. Dynamic model assays confirmed biofilm reduction with combination therapy. The combination of VAN/GEN exhibits synergistic antibiofilm activity against *S. aureus*, particularly MRSA. These findings support its potential application in catheter salvage strategies, including antibiotic lock therapy.

## 1. Introduction

Healthcare-associated infections (HAIs), especially those linked to indwelling medical devices such as central venous catheters (CVCs), continue to pose a significant public health burden [1]. Among the primary etiological agents involved, *Staphylococcus aureus* is one of the most clinically relevant pathogens due to its adaptability, virulence, and propensity to develop antimicrobial resistance [2]. In particular, methicillin-resistant *S. aureus* (MRSA) strains are notorious for their resistance profiles and have been implicated in numerous catheter-associated bloodstream infections (CRBSIs) [3]. A critical factor contributing to the persistence of these infections is the formation of biofilms.

Biofilms are structured communities of bacterial cells enclosed in a self-produced extracellular polymeric matrix that adhere to biotic or abiotic surfaces [4]. This structure confers protection against environmental stresses, immune responses, and, most importantly, antibiotic therapy. The NIH has reported that biofilms are involved in approximately 65% of all microbial infections and up to 80% of chronic infections [5]. Once established on a surface such as polyurethane—a common material in CVCs—biofilms serve as reservoirs for infection and increase the likelihood of treatment failure.

Biofilm formation on polyurethane-based medical devices poses a significant challenge in clinical settings, particularly in the context of indwelling devices such as catheters and implants. Polyurethane’s favorable mechanical properties and biocompatibility make it a common choice for medical devices; however, its surface characteristics can facilitate microbial adhesion and subsequent biofilm development [6]. Studies have demonstrated that pathogens like *S. aureus* and *S. epidermidis* readily form biofilms on polyurethane surfaces, leading to persistent infections that are difficult to treat due to the protective nature of the biofilm matrix [7]. The presence of biofilms not only complicates treatment but also increases the risk of device failure and patient morbidity. To mitigate these risks, research has focused on modifying polyurethane surfaces to resist biofilm formation. For instance, coating polyurethane with diamond-like carbon has been shown to reduce bacterial adhesion and biofilm development [8]. Additionally, incorporating quorum-sensing inhibitors into polyurethane coatings has demonstrated efficacy in disrupting bacterial communication pathways essential for biofilm maturation [9]. These advancements underscore the importance of surface engineering in the development of medical devices that are less susceptible to biofilm-associated infections.

The pathogenesis of methicillin-susceptible *S. aureus* (MSSA) and MRSA biofilms includes several steps: initial adhesion, accumulation, maturation, and dispersion. During maturation, the biofilm architecture becomes complex, harboring bacterial subpopulations in different metabolic states [2]. These microenvironments contribute to phenotypic antibiotic resistance, even in the absence of genetic resistance mechanisms. Thus, traditional antibiotics that are effective against planktonic bacteria often fail to eradicate biofilm-associated bacteria.

Vancomycin (VAN), a glycopeptide antibiotic, remains the drug of choice for MRSA infections. However, its efficacy against mature biofilms is limited due to poor penetration and the need for high concentrations to achieve bactericidal effects [10]. However, its efficacy against biofilm-associated infections remains a significant challenge. Studies have demonstrated that sub-inhibitory concentrations of vancomycin can paradoxically enhance biofilm formation in MRSA strains, potentially through the induction of membrane vesicle secretion, which facilitates bacterial adhesion and aggregation [11]. Furthermore, the biofilm environment itself can promote the emergence of vancomycin-intermediate *S. aureus* (VISA) phenotypes, driven by stress-induced genetic mutations, thereby reducing the antibiotic’s effectiveness [12]. These findings underscore the complexity of treating biofilm-associated *S. aureus* infections and highlight the need for alternative or adjunctive therapeutic strategies. Gentamicin (GEN), an aminoglycoside, has shown promise as a biofilm-disrupting agent. Its mechanism—binding to the 30S ribosomal subunit to inhibit protein synthesis—complements VAN’s inhibition of cell wall synthesis. While effective against planktonic *S. aureus* cells, gentamicin demonstrates limited activity against intact biofilms. Notably, exposure to gentamicin can paradoxically enhance biofilm biomass. In a study examining *S. aureus* biofilms on silk sutures, high concentrations of gentamicin failed to eradicate the biofilms and instead increased their biomass. This effect is attributed to the antibiotic’s inability to penetrate the biofilm matrix effectively, leading to survival and continued metabolic activity of the bacteria within [13]. These findings underscore the complexity of treating biofilm-associated infections and highlight the need for alternative or adjunctive therapeutic strategies to effectively manage such infections [13].

Although GEN is often used against Gram-negative pathogens, its synergistic potential with VAN against *S. aureus* biofilms warrants further investigation [14]. The combination of vancomycin and gentamicin has demonstrated synergistic effects against *S. aureus* biofilms, particularly when incorporated into polymethylmethacrylate (PMMA) bone cement. A study by Pedroni et al. evaluated various concentrations of vancomycin and gentamicin loaded into PMMA and assessed their efficacy in inhibiting biofilm formation [14]. The results indicated that a combination of vancomycin (4 g) and gentamicin (500 mg) significantly reduced biofilm-associated colony-forming units per milliliter (CFU/mL) compared to either antibiotic alone. Furthermore, checkerboard assays confirmed a synergistic interaction between vancomycin and gentamicin in this context. These findings suggest that co-administration of vancomycin and gentamicin in PMMA may enhance the prevention of biofilm-related infections in orthopedic applications.

This study aims to evaluate the efficacy of VAN and GEN, alone and in combination, against biofilms formed by methicillin-sensitive and methicillin-resistant *S. aureus* strains on polyurethane surfaces. By employing both static and dynamic in vitro models, we seek to simulate real-world catheter conditions and identify therapeutic combinations that may enhance clinical outcomes.

## 2. Materials and Methods

### 2.1. Ethical Considerations and Study Design

**Figure 1:**
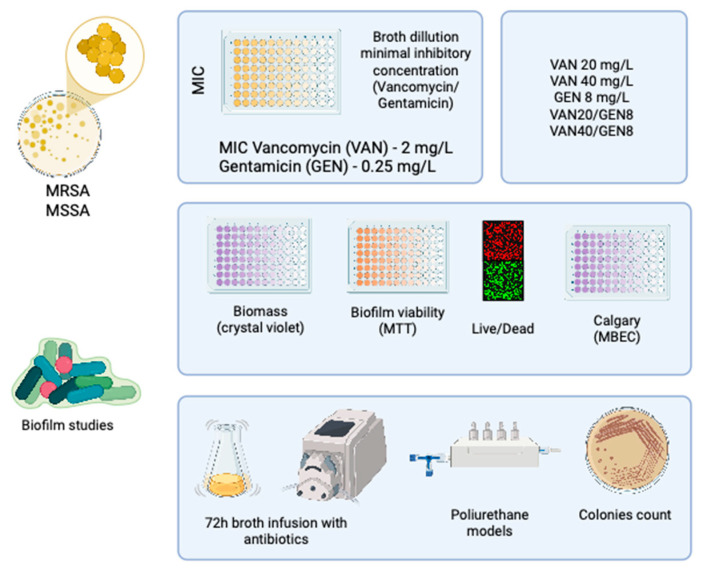
Scheme of the biofilm experiment with MRSA and MSSA, including minimal inhibitory concentration (MIC) analysis, biomass, and viability of the biofilm with different doses of vancomycin and gentamicin. The figure also shows a live/dead immunofluorescence assay used to evaluate biofilm viability and a dynamic model with polyurethane models with 72 h of antibiotics continuous infusion.

This study was conducted in compliance with ethical standards and was approved by the Ethics Committee of Pontifical Catholic University of Paraná (PUCPR) under protocol number 28859719.3.0000.0020. It was designed as an experimental, in vitro investigation evaluating the antibiofilm activities of VAN and GEN, both individually and in combination, against *S. aureus* biofilms formed on polyurethane surfaces (Figure 1).

### 2.2. Bacterial Strains and Identification

A clinical isolate of MRSA was obtained from positive blood cultures collected at Hospital Universitário Cajuru and MSSA ATCC 25923 were used in the study. The isolates were cultured on tryptic soy agar (TSA) plates (Kasvi, Pinhais, Brazil) and incubated overnight at 37 °C. Identification was confirmed using MALDI-TOF mass spectrometry (Bruker Microflex LT, Bremen, Germany). Isolates were preserved in tryptic soy broth (TSB) supplemented with 15% glycerol (Kasvi, Pinhais, Brazil) at −80 °C for future use.

### 2.3. Antimicrobial Agents and Preparation

Vancomycin hydrochloride (Blau Farmaceutica, Cotia, Brazil) and gentamicin sulfate (Santisa, Brazil) were used. Stock solutions were prepared based on CLSI M07-A10 guidelines: VAN at 50 mg/mL and GEN at 256 mg/L, stored under sterile conditions at appropriate temperatures.

### 2.4. Determination of Minimum Inhibitory Concentration (MIC)

MICs of VAN and GEN were determined using the broth microdilution technique as recommended by CLSI guidelines [15]. Briefly, bacterial suspensions were standardized to 3 × 10^5^ CFU/mL in Mueller-Hinton broth (MHB) (Kasvi, Pinhais, Brazil). Serial two-fold dilutions of each antibiotic were prepared in 96-well microtiter plates. After 24 h of incubation at 37 °C, MIC was defined as the lowest concentration that inhibited visible bacterial growth. All experiments were conducted in triplicate.

### 2.5. Evaluation of Growth Inhibition in Combination Therapy

For subsequent assays, groups were organized with vancomycin at 20 mg/L (VAN20), 40 mg/L (VAN40), gentamicin at 8 mg/L (GEN), and two combination groups pairing VAN20 and VAN40 with GEN. These concentrations were selected based on variations commonly reported for clinical and microbiological control in bloodstream infections [16,17,18,19,20]. VAN20 reflects a clinically achievable therapeutic concentration, whereas VAN40 represents a higher target for microbiological eradication. The GEN concentration was set at 10× the median MIC, derived from an estimated standard dose of 1 mg/kg [21,22].

To assess the inhibitory effects of VAN and GEN combinations, macrodilution assays were performed using tubes containing 1 mL of MHB inoculated with *S. aureus* at 3 × 10^5^ CFU/mL. Antibiotics were added at final concentrations of VAN 20 and 40 µg/mL, and GEN at 4 and 8 µg/mL. Combination groups included VAN + GEN at both concentrations. After incubation at 37 °C for 24 h, bacterial turbidity was visually assessed, and 100 µL aliquots from non-turbid tubes were plated on TSA and incubated for quantification.

### 2.6. Biofilm Formation and Quantification

Biofilms were formed on sterile 96-well microtiter plates. Bacterial suspensions of approximately 1 × 10^7^ CFU/mL in TSB were added to the wells and incubated at 37 °C for 24 h [23]. The supernatant was discarded and washed three times with NaCl 0.9% (Equiplex, Goiania, Brazil). Then, VAN40, VAN20, VAN40/GEN, and VAN20/GEN were tested during a 24 h period in the biofilm incubated at 37 °C. A negative control (only TSB) and positive control (ethanol 70% (Equiplex, Goiania, Brazil)) were included.

Biofilm biomass was assessed using the crystal violet (CV) retention assay (Sigma-Aldrich, Darmstadt, Germany). After 24 h with antibiotics, the biofilms were fixed with methanol (Equiplex, Goiania, Brazil), stained with 1% CV, rinsed, and destained with 33% acetic acid (Sigma-Aldrich, Darmstadt, Germany). Absorbance was measured at 480–570 nm using a microplate reader. Each experiment was performed in triplicate.

To evaluate biofilm metabolic activity, MTT assays were performed [24]. After biofilm development and treatment, 200 µL of MTT solution (1 mg/mL) (Sigma-Aldrich, Darmstadt, Germany) was added to each well and incubated for 4 h at 36 °C. The supernatant was discarded, and isopropanol (Equiplex, Goiania, Brazil) was added to dissolve the formazan crystals. The absorbance at 570 nm was measured as an indicator of viable cell activity. All experiments were performed in triplicate.

### 2.7. Minimum Biofilm Eradication Concentration (MBEC) Assay

A modified Calgary Biofilm Device method was used. Biofilms were grown on pin-lid polyurethane (Basf Tpu 95 A, Heidelberg, Germany) devices in 96-well plates [25]. Each peg of the device has an approximate surface area of 109 mm^2^; its rounded “tip” extends approximately 4 to 5 mm into the growth medium. The surface of the polyurethane is ultra-smooth. After 24 h incubation, pins were transferred to plates containing serial dilutions of antibiotics (VAN 16,384 to 8 mg/L; GEN 4096 to 2 mg/L; and combinations). Following a 24 h treatment, pins were washed, transferred to TSA plates, and incubated. MBEC was defined as the lowest concentration preventing visible regrowth.

### 2.8. Live/Dead Fluorescence Microscopy

Biofilms formed in 8-well chambers were treated and stained using the FilmTracer™ LIVE/DEAD^®^ Biofilm Viability Kit (Thermo Fisher Scientific, Waltham, MA, USA). SYTO9 and propidium iodide were used to differentiate live and dead cells, respectively. After staining, samples were examined under a Leica DM fluorescence microscope.

This method utilizes two nucleic acid stains: SYTO 9, which penetrates all bacterial membranes and stains both live and dead cells green, and propidium iodide, which selectively penetrates cells with compromised membranes, displacing SYTO 9 and emitting red fluorescence. As a result, live bacteria appear green, while dead or membrane-damaged cells fluoresce red. When analyzed using confocal laser scanning microscopy, the kit enables both qualitative and quantitative assessment of biofilm viability, offering insight into the spatial distribution and proportion of live and dead bacterial populations within the biofilm matrix [26]. Image analysis was performed using LAS software, Version 5.3.0.

### 2.9. Dynamic Biofilm Model Under Continuous Flow

A peristaltic pump system was employed to simulate catheter flow conditions. Five sterile polyurethane pins (for each group) were pre-inoculated and placed in flow chambers connected to a reservoir of TSB supplemented with VAN, GEN, or their combinations. The flow rate was set at 1 mL/min for 72 h. Post-incubation, pins were removed, sonicated in saline, and CFUs were determined by serial dilution and TSA plating.

### 2.10. Statistical Analysis

All assays were performed in biological and technical triplicates. Data were analyzed using GraphPad Prism 7.0. Quantitative data were expressed as medians with interquartile ranges. Mann–Whitney U and Kruskal–Wallis tests followed by Dunn’s multiple comparisons test were used. Statistical significance was defined as *p* < 0.05.

## 3. Results

### 3.1. Minimum Inhibitory Concentration (MIC) Results

The MICs for VAN and gentamicin GEN were determined. Both isolates exhibited MIC values of 2 µg/mL for VAN and 0.25 µg/mL for GEN, which were consistent across replicates. These values were within expected ranges for *S. aureus* susceptibility, confirming strain sensitivity prior to biofilm assessments.

### 3.2. In Vitro Growth Inhibition by Monotherapy and Combination Therapy

Macrodilution assays revealed that VAN at 40 mg/L completely inhibited growth of all tested *S. aureus* strains, both MRSA and MSSA. GEN alone at 4 mg/L showed limited inhibition, while 8 mg/L significantly reduced turbidity, particularly in MSSA isolates. Combination therapy with VAN 20 mg/L + GEN 8 mg/L or VAN 40 mg/L + GEN 8 mg/L consistently prevented visible growth. Colony counting confirmed the synergistic inhibition, especially in MRSA strains, where GEN alone was ineffective.

### 3.3. Biofilm Biomass Quantification and MTT

**Figure 2:**
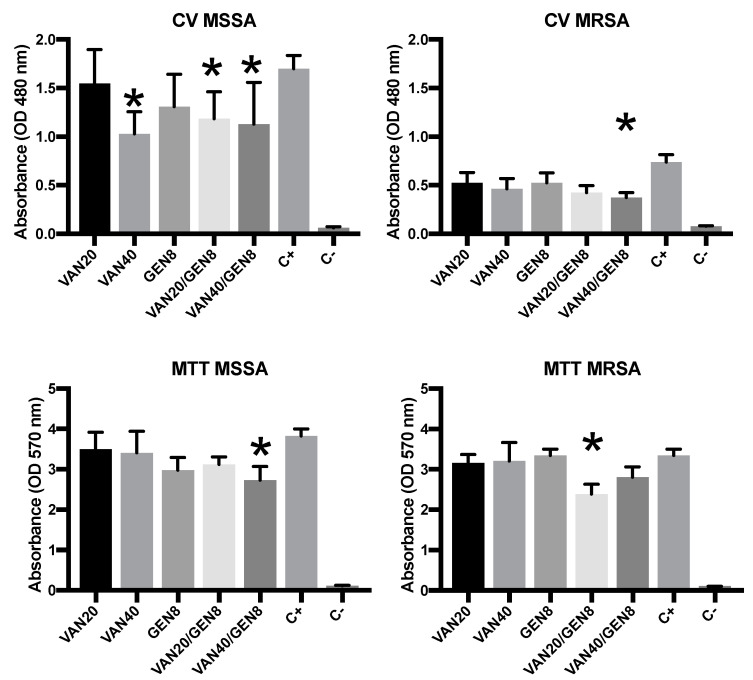
Biomass (CV) and MTT of different doses and combinations of vancomycin (VAN) and gentamicin (GEN) in MRSA and MSSA isolates. C+ = positive control; C− = negative control. * *p* value < 0.05 in comparison with positive control.

Crystal violet (CV) staining demonstrated that MSSA biofilms were significantly reduced with monotherapy at higher VAN concentrations (40 mg/L), while GEN alone showed moderate reduction. MRSA biofilms were more resistant; VAN or GEN alone had minimal effect on biomass, but the combination of VAN20/GEN8 reduced MRSA biomass by over 60% (*p* < 0.01). The most pronounced reduction was observed with VAN40/GEN8, indicating synergistic action (Figure 2).

MTT assays indicated similar trends. In MSSA, VAN alone caused a significant reduction in metabolic activity, while GEN’s effect was modest. In MRSA, metabolic activity remained high under monotherapy, suggesting active cells persisted within the biofilm matrix. However, combined therapy (VAN20/GEN8) decreased MRSA metabolic activity by over 70% (*p* < 0.01). This confirms that combination therapy not only reduces biomass but also compromises cellular viability within biofilms.

### 3.4. Minimum Biofilm Eradication Concentration (MBEC)

The MBEC assays provided critical insight into the concentrations required to eradicate established biofilms. For MSSA, gentamicin eradicated the biofilm at 256 mg/L, and vancomycin do not achieved biofilm eradication, even with 16,385 mg/L. For MRSA, gentamicin eradicated the biofilm at 256 mg/L, and vancomycin at 1024 mg/L. The combination therapy at VAN40/GEN8 or VAN20/GEN8 led to no observable regrowth on MRSA, representing complete eradication. However, for MSSA, the combination showed no efficacy. These MBEC values suggest that monotherapies are insufficient for biofilm eradication, and support the use of combination, at least for MRSA.

### 3.5. Fluorescence Microscopy Imaging of Biofilm Viability

**Figure 3:**
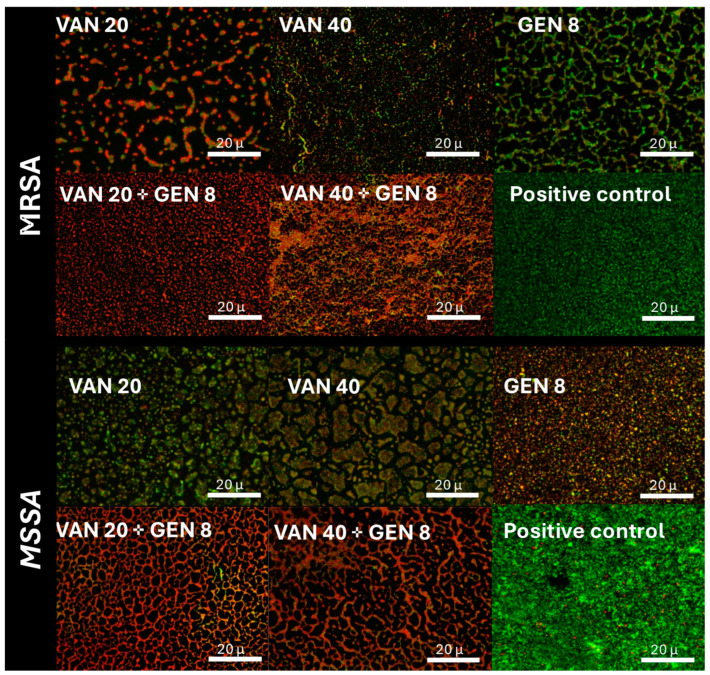
Live/dead analysis by immunofluorescence assay to evaluate the viability of sessile cells on biofilm. Red is considered dead cells, and green is considered viable cells. MRSA and MSSA isolates. The combinations of vancomycin (VAN) and gentamicin (GEN) suggest a better activity.

Fluorescence microscopy using SYTO9/PI staining revealed predominance of green fluorescence (live cells) in untreated and monotherapy groups, especially in MRSA. MSSA biofilms treated with VAN showed a modest increase in red fluorescence (dead cells). The combination of VAN and GEN yielded pronounced red fluorescence in MRSA and MSSA biofilms, supporting the bactericidal effect of the dual therapy (Figure 3).

### 3.6. Evaluation of Antimicrobial Activity in the Dynamic Biofilm Model

**Figure 4:**
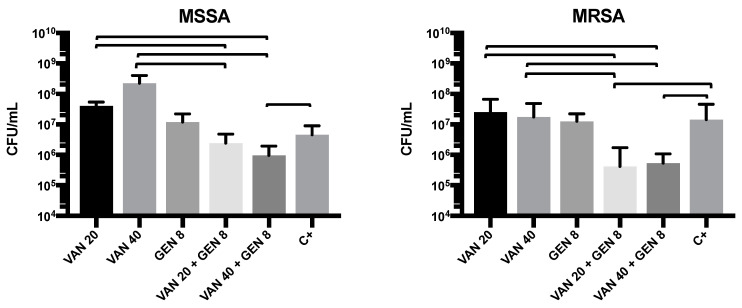
Colony-forming unit (CFU) counts in dynamic biofilm under 72 h continuous infusion of vancomycin (VAN) and/or gentamicin (GEN) at different concentrations against MRSA and MSSA isolates. Lines indicate statistical difference in each group (*p* < 0.05).

In the continuous-flow model simulating catheter conditions, biofilms developed over 72 h on polyurethane pins showed consistent colonization in both MRSA and MSSA. CFU enumeration after treatment revealed that monotherapy with VAN40 or GEN8 caused only partial reduction in MRSA biofilms (1–2 log reduction). MSSA was more susceptible to GEN alone. However, the combination of VAN20/GEN8 led to a 4-log CFU reduction in MRSA and 2-log reduction in MSSA (*p* < 0.001). These findings demonstrate that vancomycin and gentamicin act synergistically to disrupt and eradicate biofilms formed by *S. aureus* on polyurethane—a material widely used in medical devices. The superiority of combination therapy was evident across static and dynamic conditions (Figure 4).

## 4. Discussion

The present study demonstrates that the combination of VAN and GEN provides a synergistic effect against *S. aureus* biofilms, particularly methicillin-resistant strains, when tested on polyurethane surfaces that simulate intravascular catheters. These findings reinforce the therapeutic potential of dual antibiotic regimens in the management of catheter-related bloodstream infections, especially in scenarios involving biofilm-producing pathogens. This strategy can also be used to salvage intravascular catheters, allows the localized administration of high-dose antibiotics to eradicate biofilms while minimizing systemic toxicity (lock therapy) [27,28].

Lock therapy, also known as antibiotic lock therapy (ALT), is a targeted approach employed to prevent and treat catheter-related bloodstream infections, particularly those associated with biofilm-forming microorganisms [29]. The technique involves instilling a high-concentration antimicrobial solution into the catheter lumen, which is then “locked” in place for an extended period, typically several hours, without being flushed through the bloodstream. This method aims to eradicate pathogens embedded within the biofilm matrix on the internal surfaces of catheters, a common source of persistent infections. ALT is especially beneficial when catheter removal is not feasible, offering a means to salvage the device while effectively managing the infection. The success of this therapy hinges on the appropriate selection of antimicrobial agents, their concentration, and sufficient dwell time to ensure efficacy against the targeted pathogens [29].

The combination of vancomycin and gentamicin in lock therapy has shown promising results for the treatment and prevention of catheter-related infections, particularly those caused by Gram-positive organisms such as *S. aureus* and coagulase-negative *Staphlylococci*. Vancomycin targets the bacterial cell wall, while gentamicin interferes with protein synthesis, and their combined use can enhance antimicrobial efficacy, including activity against biofilm-embedded bacteria. Studies have demonstrated that using vancomycin and gentamicin together in lock solutions results in greater reductions in bacterial load compared to either agent alone, due to their synergistic effect [30]. This combination is particularly useful when treating polymicrobial infections or in cases involving multidrug-resistant strains. Additionally, the inclusion of anticoagulants such as heparin or citrate in the lock solution can help prevent catheter occlusion and enhance drug stability. Therefore, vancomycin–gentamicin lock therapy represents an effective strategy to manage persistent intraluminal infections while preserving vascular access.

Vancomycin, although a gold standard for MRSA treatment, has long been criticized for its suboptimal performance in biofilm-associated infections [31]. This limitation is largely due to the physicochemical properties of biofilms, which hinder antimicrobial penetration and limit drug exposure to the bacterial cells embedded in the matrix [9]. Gentamicin, while not traditionally used for monotherapy in Gram-positive infections, can act as a synergistic agent by disrupting the extracellular polymeric substances [32,33].

Our results demonstrate that while VAN or GEN alone had modest effects in static conditions, their combination led to significant biomass reduction, lowered metabolic activity, and even complete eradication in some MRSA biofilms. Notably, the metabolic assays (MTT) and biomass measurements (CV) were complementary and revealed that monotherapy with GEN at standard concentrations did not substantially impair the biofilm. However, when paired with VAN, metabolic activity was dramatically decreased. Other study showed that combining GEN with quercetin enhances the antibacterial activity, disrupts *S. aureus* cell membrane integrity, and increases reactive oxygen species production, leading to enhanced bacterial cell lysis [34]. Thise strategy also inhibits biofilm formation, reduced viable cell counts, and diminished the extracellular matrix components [35].

In fluorescence microscopy, the red fluorescence observed in MRSA biofilms treated with both VAN and GEN confirms enhanced cell death, correlating with the MBEC results that showed eradication in these conditions. This reinforces the idea that GEN facilitates access of VAN to deeper biofilm layers, leading to bactericidal activity that neither agent could achieve alone. Studies evaluating the combination of vancomycin and gentamicin are relatively scarce in the literature. However, gentamicin has demonstrated synergistic effects when combined with other antimicrobial agents, particularly in the context of biofilm-associated infections. It is worth noting that the concentrations of aminoglycosides required to achieve antibiofilm activity are often higher than those used in standard systemic therapy [36,37].

Importantly, the dynamic flow system developed for this study simulates clinical catheter conditions more accurately than static assays. In this system, the combination therapy again outperformed monotherapy, resulting in 4-log reductions in MRSA biofilm viability. This finding underscores the clinical relevance of such models in evaluating therapeutic efficacy, as biofilms in the bloodstream are constantly exposed to shear forces and nutrient flow that are absent in static plates [38,39].

The differential responses between MRSA and MSSA in this study are also notable. While MSSA was moderately affected by monotherapy with VAN, eradication required combination treatment, illustrating the phenotypic plasticity and biofilm tolerance inherent to both strains [40,41]. These findings suggest that the susceptibility of planktonic cells does not reliably predict biofilm eradication, a conclusion echoed in multiple recent studies [42,43].

A major clinical implication of these findings is the potential role of combination therapy in catheter salvage strategies [44]. Currently, catheter removal is often the preferred intervention for CRBSIs. However, in situations where removal is contraindicated or impractical, local antibiotic lock therapy using synergistic agents like VAN and GEN may serve as an alternative. This is particularly relevant in immunocompromised or pediatric patients where catheter access is limited [45]. Although the study was conducted using polyurethane, it is important to consider the applicability of this type of research to other materials, since this polymer is not the only one used in the manufacture of catheters and other implantable devices [46]. Biofilm formation may vary depending on the material, which could influence the performance of the combined regimen of vancomycin and gentamicin [47].

The limitations of our study include its in vitro nature, which cannot fully replicate the host immune response or pharmacokinetic variables present in vivo. Additionally, while the dynamic model approximates clinical flow, it does not include serum proteins or immune mediators that could alter biofilm physiology. Nevertheless, the reproducibility and consistency of our data across assays support the robustness of the observed effects.

Future studies should focus on confirming these findings in vivo, potentially using animal catheter models or human clinical trials. Moreover, investigating the efficacy of other aminoglycosides or glycopeptide alternatives could further refine treatment protocols. The future of combination therapy with vancomycin and gentamicin for the treatment of biofilm-associated infections is focused on enhancing drug delivery, improving biofilm penetration, and reducing toxicity. Although the synergistic action of these antibiotics has shown efficacy against pathogens such as *S. aureus*, their limited ability to eradicate mature biofilms remains a significant challenge due to restricted diffusion and bacterial tolerance mechanisms. Emerging strategies include the use of nanocarrier systems—such as liposomes, nanoparticles, and hydrogels—that can enhance targeted delivery and sustained release of the drugs within the biofilm matrix. Additionally, the incorporation of these antibiotics into antimicrobial coatings or lock solutions for medical devices, particularly when combined with biofilm-disrupting agents, may help prevent biofilm formation. The use of adjunctive therapies, including quorum-sensing inhibitors, dispersal enzymes, and metabolic adjuvants, also holds promise in increasing bacterial susceptibility. Finally, advances in personalized medicine may allow for customized lock therapy protocols based on pathogen characteristics and biofilm maturity, ultimately improving clinical outcomes.

## 5. Conclusions

In conclusion, this work provides compelling evidence that vancomycin and gentamicin in combination have superior antibiofilm activity compared to either agent alone. Their synergistic effect is evident in both static and dynamic conditions, suggesting that this therapeutic approach may enhance outcomes in biofilm-associated *S. aureus* infections, especially when catheter preservation is essential. The findings of this study reinforce the critical need for combination antibiotic therapy in the management of device-related infections caused by *S. aureus*. Vancomycin and gentamicin together showed robust synergistic activity, effectively reducing biomass and metabolic viability, and achieving eradication under dynamic flow conditions. These results, validated across multiple in vitro systems, offer a promising strategy for improving outcomes in catheter-associated bloodstream infections.

## Data Availability

Data are available under request.
